# Lifestyle factors and oncogenic papillomavirus infection in a high-risk male population

**DOI:** 10.1371/journal.pone.0184492

**Published:** 2017-09-12

**Authors:** Elena Lopez-Diez, Sonia Perez, Manuel Carballo, Amparo Iñarrea, Angel de la Orden, Maximo Castro, Moises Rodríguez, Sheila Almuster, Ruben Montero, Miguel Perez, Jorge Sanchez, Antonio Ojea

**Affiliations:** 1 Department of Urology, University Hospital of Vigo, Vigo, Spain; 2 Department of Microbiology. University Hospital of Vigo, Vigo, Spain; 3 Department of Obstetrics and Gynecology. University Hospital of Vigo, Vigo, Spain; Rudjer Boskovic Institute, CROATIA

## Abstract

**Background:**

High risk human papillomavirus (HR-HPV) infection in males is a health issue with implications for HPV-related lesions in their partners. The identification of risk factors for male infection may improve our understanding of HR-HPV transmission and prevention. The aim of this study was to evaluate the relationships between lifestyle, genital warts and HR-HPV infection. The study was focused on men with an increased risk of HR-HPV infection: male sexual partners of women diagnosed with high-grade squamous intraepithelial cervical lesions.

**Methods:**

Men were enrolled and prospectively recruited within the first six months after diagnosis of cervical lesions in their female partners (n = 175, 2013–2016). Epidemiological and sexual behaviour data were obtained. The presence of genital warts was established by visual inspection. Detection and genotyping of HR-HPV infection in genital samples were performed with a Linear Array HPV Genotyping Test. All HR-HPV positive men were offered a follow-up exam at 12 months. SPSS version 19 was used for statistical analysis.

**Results and discussion:**

The prevalence of HR-HPV infection in men was 45.1% (79/175). Genital warts were observed in 10.3% (18/175) of the subjects. Detection of genital warts (OR 3.5, *p* = 0.015), smoking habits (OR 2.3, *p* = 0.006) and sexual debut before 16 years old (OR 2, *p* = 0.035) were associated with an increased risk for HR-HPV infection (univariate analysis). This association was also observed for genital warts and smoking status in a multivariate analysis. The same genotype was found after one year in 71.4% (20/28) of subjects.

**Conclusions:**

The presence of genital warts and smoking habits seem to be associated with a higher risk of HR-HPV infection in males. Earlier sexual debut may increase this risk. Extensive knowledge of the natural history of HR-HPV infection in males is an absolute requirement for the design and implementation of prevention strategies for the general population as well as for specific populations such as couples after treatment for high-grade cervical lesions.

## Background

Human papillomavirus (HPV) infection is one of the most common sexually transmitted infections. It is known that up to 70% of both men and women of the world population will be infected during their lifetime. Approximately 60 HPV genotypes are involved in infection of the genital tract, and at least 12 genotypes are considered to be high risk (HR-HPV) or oncogenic [1,2] with different carcinogenic potential depending on the HR-HPV genotype.

The main consequence of HR-HPV infection is cervical cancer, being which is one of the leading causes of cancer-related mortality in women [3]. This relationship accounts for 99.7% of cervical cancer worldwide and 96.8% in our community, as described in detail previously [4]. Moreover, previous studies suggested that cancers of penis and cervix may share the same aetiological factors [5]. Although rare, penile cancer is associated with a high morbidity and mortality. The carcinogenesis of penile cancer is thought to involve two pathways: one related to inflammation and other dermatological conditions of the penis, and other related to HPV infection [6]. About one-third to one-quarter of invasive penile cancers may be related to HR-HPV according to retrospective studies [7]. Male infection is usually asymptomatic [8,9], and this could explain why the incidence of HR-HPV associated cancers and pre-cancerous lesions are now increasing.

Since the risk of HPV infection and cervical cancer in females is significantly influenced by male behaviour [10,11], a greater understanding of the natural history of HPV infection in men is critical for cervical cancer prevention. Although there are no current recommendations regarding screening programmes for asymptomatic infection in men [12], investigations of the presence of HPV in men who are sexual partners of infected women are becoming more common.

Some of the most consistently reported risk factors for the acquisition of HPV infection in men are higher number of current and past sex partners (SP) [13], lack of male circumcision (MC) [14], younger age at first sexual intercourse (FSI) [15], lack of condom use [9], having a sex partner with high-grade squamous intraepithelial cervical lesions (HGLC) [16] and smoking status [17].

The strength of the reported associations vary depending on the features of the populations under examination and the HPV genotype involved, i.e., oncogenic, non-oncogenic or both.

Since it is known that HPV vaccination is a potential preventive approach for men, additional insight from epidemiological studies of HPV infection in men is needed.

The aim of this study was to establish relationships between lifestyle factors, genital warts (GW) and HR-HPV infection in a high-risk population: male sexual partners of women diagnosed with high-grade squamous cervical lesions.

## Materials and methods

### Study population

A cross-sectional study was conducted by the Urology Department of the University Hospital of Vigo. Between January 2014 and December 2016, 202 asymptomatic men older than 18 years were invited to participate in this study because their current partners (regular sexual intercourse for more than 1 year) presented HGCL (cervical intraepithelial neoplasia grade 2 or grade 3-carcinoma in situ) in the previous six months. These couples were selected from the Cervical Cancer Screening Consulting, Department of Obstetrics and Gynaecology, Vigo, Spain. Only 175 of 202 (86.6%) couples met the inclusion criteria for the study. Women were diagnosed by cytology, colposcopy and histological examination. HPV genotypes associated with lesions in women were found in 171 cases. After physical examination, a penile scraping was obtained and submitted to PCR assay to identify HPV carriers. The institutional review board approved the study (cod 2013/470 from the ethics committee of clinical investigation of Galicia Santiago de Compostela, Spain). Information concerning the research project was provided to all participants. Written informed consent was obtained from all couples. A genital examination was done to identify signs of flat, macular and acuminate lesions (GW). Men were invited to fill in a self-administered questionnaire on lifestyle, including sexual behaviour (SB). Because most men are not knowledgeable about the implications of a positive HPV test, the staff spent the necessary time educating men about HPV. We explained that a positive test for the virus does not necessarily put them at risk for disease. Participants did not receive any incentive for study involvement.

All HR-HPV positive men were invited to a clinical follow-up at 12 months. Condom use was recommended to all couples. Women were advised to take the HPV vaccine in accordance with Spanish recommendations to prevent new infections by different HR-HPV genotypes [18]. All women were treated for their HR-HP- related lesions.

### Specimen collection

Three, dry cytobrushes for each male were used to scrape the genitalia three times to collect exfoliated cells from different penile areas, including the dorsal and ventral penile area, external and internal surface of prepuce, coronal sulcus, glans and distal urethra. The three samples were combined and collected into one single vial containing molecular biology grade TE buffer pH 8.0 (AppliChem GmbH, Darmstadt, Germany). Samples were maintained at 2–8°C and processed within 24–72 hours after collection.

### Epidemiological survey

The following variables were studied: age, nationality, HR-HPV infection and specific HPV genotype, age at first sexual intercourse (FSI), number of sexual partners (SP) in the year preceding the study, number of sexual partners up to the date of the study, smoking in the last year, circumcision, regular condom use in the last year, and presentation of genital warts. An extended questionnaire (S1 Appendix) was validated in a previous study by our team [4] with the assistance of a research statistician, and only those questions considered the most interesting for this study and population were selected.

### HPV DNA detection and genotyping

DNA was isolated using the QIAamp MinElute Media Kit (Qiagen, Hilden, Germany). The extracted nucleic acids were stored at -20°C. An aliquot of the original sample was also stored at -20°C. Amplification and detection were carried out using the Linear Array HPV Genotyping Test (Linear Array. Roche Diagnostics, Mannheim, Germany) according to the manufacturer's instructions. We described the distribution of 21 HR-HPV genotypes classified as HR (HR-HPV, IARC Group 1 carcinogens) or probable/possible HR (pHR-HPV, IARC Group 2A/B carcinogens) (Table 1) by the International Agency for Research on Cancer Monograph Working Group [1]. HPV types 30, 85, and 97 were not included in this study because the genotyping test used in this study does not allow their detection. We also described the distribution of 2 low-risk HPV genotypes involved in genital warts (HPV 6 and 11). This test also detects human beta-globin to control the sample adequacy and quality in addition to success in DNA extraction and PCR (internal control).

**Table 1:** HPV genotypes detected in this study.

IARC classification	HPV genotypes
HR-HPV	16, 18, 31, 33, 35, 39, 45, 51, 52, 56, 58, 59
pHR-HPV	26, 34, 53, 66, 67, 68, 69, 70, 73, 82
LR-HPV	6, 11

IARC: International Agency for Research on Cancer. HR-HPV: high-risk HPV genotypes; pHR: probable/possible high-risk genotypes, LR: low-risk genotype. V. Bouvard [1]

### Statistical analysis

Age and standard deviation were calculated. Qualitative variables were compared using the Chi-square test. The *odds* ratio (OR) and corresponding 95% confidence interval (95% CI) were calculated. A *p* value <0.05 was considered statistically significant. Multivariate logistic regression analysis was used to evaluate factors that were independently associated with HR-HPV detection and with 12 months persistence of HR-HPV infection in males (step-wise logistic regression models). Only variables with *p* ≤0.25 in the bivariate analysis were included in the multivariate analysis. A manual stratified analysis was conducted to explore potential interactions and additive effects among variables. SPSS version 19.0 (Statistical Package for Social Sciences, Chicago, IL, USA) was used for statistical analysis.

## Results

**Fig 1:**
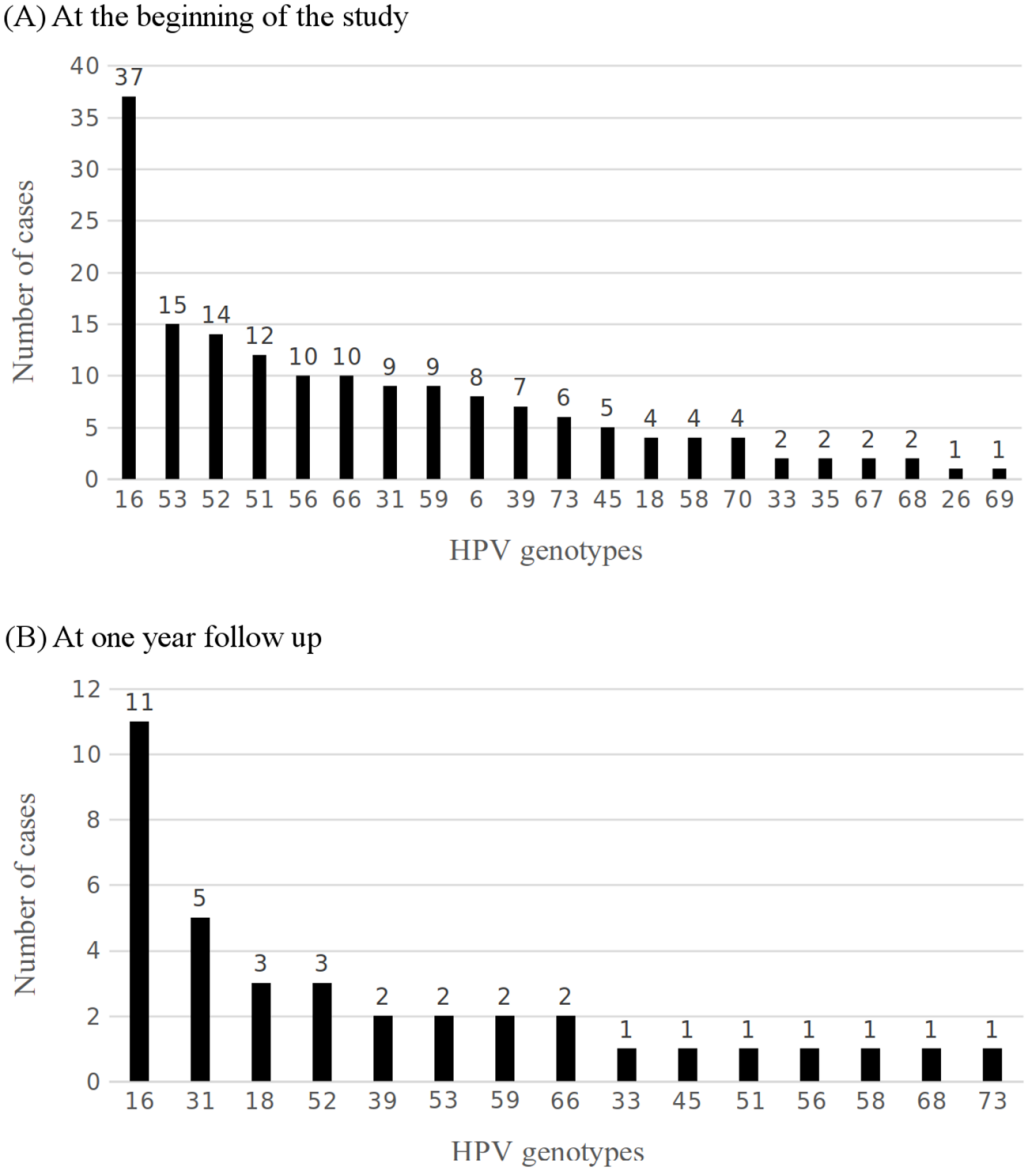
HPV genotype distribution. (A) Number of cases shown for each HPV genotype at the beginning of the study. (B) Number of cases shown for each HPV genotype after one year follow up.

A total of 175 couples were included. The average age of men was 38.6 ± 8.7 (22–70) years. The average age of women was 36.1 ± 8.0 (22–65) years. The prevalence of HR-HPV infection was 45.1% (79/175) in men and 97.7% (167/171) in their female partners. Their distribution is shown in Fig 1A. HPV16 was the most frequent genotype and was detected in 21.1% (37/175) of infected men. Multiple HR-HPV infections were detected in 26.3% (46/175) of men (for the distribution see S1 Table). Genital warts were diagnosed in 10.3% (18/175). The epidemiological characteristics are described in Table 2.

**Table 2:** Sociodemographic, lifestyle and sexual behaviour characteristics of participants.

	Men (n = 175)
Age (mean ± SD); years	38.6 ± 8.7
HPV +	79 (45.1%)
HPV 16 +	37 (21.1%)
Multiple HPV +	46 (26.3%)
**First sexual intercourse**	
*> 16 years*	108 (61.7%)
*≤ 16 years*	67 (38.3%)
**Lifetime sexual partners**	
*≤ 5 sexual partners*	40 (22.9%)
*> 5 sexual partners*	135 (77%)
**Recent sexual partners**	
1 *sexual partners*	149 (85.1%)
> 1 *sexual partners*	26 (14.9%)
**Male circumcision**	
Yes	23 (13.1%)
No	152 (86.9%)
**Condom use**	
*Yes*	67 (38.3%)
*No*	108 (61.7%)
**Genital warts**	
*Yes*	18 (10.3%)
*No*	157 (89.7%)
**Smoking status**	
*Yes*	93 (46.9%)
*No*	82 (53.1%)

SD: Standard Deviation; HPV: Human Papillomavirus infection

Smoker status (54.8% (51/93) *vs*. 34.1% (28/82), OR 2.3; 95%CI: 1.2–4.3) and the presence of genital warts (77.2% (13/18) *vs*. 42% (66/157), OR 3.5; 95%CI: 1.2–10.5) were associated with an increased risk for HR-HPV infection in men in the univariate analysis. Being under 16 years old at first sexual intercourse was also associated with an increased risk for HR-HPV infection in men (Table 3). Male circumcision, condom use, lifetime sexual partners and monogamous relationship with an HPV-infected woman in the last year were factors that were not associated with an increased risk for HR-HPV infection. In the multivariate analysis, genital warts (OR 3.9, 95% CI: 1.3–11.9) and smoking habit (OR 2.4, 95% CI: 1.3–4.6) were associated with a higher risk for HR-HPV infection in this male population (Table 3).

**Table 3:** Bivariate and multivariate odds ratios for the associations of lifestyle and sexual behaviour characteristics with genital HPV prevalence among male partners of women recently diagnosed with high-grade cervical lesions.

	HPV+ men		Bivariate		Multivariate	
	Yes (n = 79)	No (n = 96)	OR_crude_ (95%CI)	p-value	OR_adjusted_ (95%CI)	p-value
Age at first sexual intercourse; n (%)						
<16 years	42 (38.9)	66 (61.1)	1.93 (1.05–3.59)	0.035		
>16 years	37 (55.2)	30 (44.8)				
Lifetime sexual partners; n (%)						
<5 sexual partners	14 (35.0)	26 (65.0)	1.72 (0.83–3.59)	0.142		
>5 sexual partners	65 (48.1)	70 (51.9)				
Recent sexual partners; n (%)						
1 sexual partners	65 (43.6)	84 (56.4)	1.51 (0.65–3.48)	0.334		
>1 sexual partners	14 (53.8)	12 (46.2)				
Genital warts; n (%)						
Yes	13 (72.2)	5 (27.8)	3.58 (1.22–10.5)	0.015	3.94 (1.30–11.9)	0.015
No	66 (42.0)	91 (58.0)				
Condom use; n (%)						
Yes	31 (46.3)	36 (53.7)	1.08 (0.58–1.99)	0.814		
No	46 (44.4)	60 (55.6)				
Male circumcision; n (%)						
Yes	9 (39.1)	14 (60.9)	1.33 (0.54–3.25)	0.534		
No	68 (46.1)	82 (53.9)				
Smoking status; n (%)						
Yes	49 (54.8)	42 (45.2)	2.34 (1.27–4.32)	0.006	2.47 (1.32–4.62)	0.005
No	28 (34.1)	54 (65.9)				

OR: Odds Ratio; HPV: Human Papillomavirus infection; CI: Confidence interval

A total of 55 HR-HPV positive patients fulfilled criteria for a one-year follow-up. All HPV positive men received a letter of reminder for the follow-up date. Among them, 28 (50.9%) agreed to complete the follow-up and attended the scheduled appointment. Mean age, HPV16 infection and severity of female histological alterations were similar between those who agreed to complete the follow-up and those who did not. The same HR-HPV genotype was found in 71.4% (20/28). HPV16 was found in 55% (11/20) of patients with the same genotype infection at the 12-month follow-up. Their distribution is shown in Fig 1B. Being under 16 years old at first sexual intercourse (*p* = 0.686), smoking status (*p* = 0.615), genital warts (*p* = 0.311), male circumcision (*p* = 0.503), condom use (*p* = 1), lifetime sexual partners (*p* = 1) and monogamous relationship with an HPV-infected woman in the last year (*p* = 0.295) were not associated with an increased risk for HR-HPV infection at the one-year follow-up.

## Discussion

The objective of this study was to assess the risk factors associated with HR-HPV infection in a high-risk male population. Male SPs of women with HGCL have been the focus of previous studies [8,19–21]. In our population, the prevalence of HR-HPV infection was high (i.e., almost half of the studied cases); this result is in agreement with data reported in similar studies (30%-68%) [8,22]. Polymerase chain reaction (PCR) is well established as the most sensitive detection method considering that there is no licensed test for HPV detection in men [20,23]. Because every woman was proven to be infected by HR-HPV in the previous months, all their male partners were exposed to the virus.

It is still unclear whether a reinfection pattern between couples could occur [4]. It was stated that some immune protection from viral antibodies induced by past exposure would result in less overall detection of incidental infection among long-lasting couples [24].

In addition to HPV16, which is the most common genotype [25], other genotypes were also involved in both single infection or co-infection with HPV16. The prevalence pattern for specific genotypes in men is still unclear [26]. Several studies have shown that the eight most prevalent HPV types in cervical cancer are geographically stable, with HPV16 being the most common [2].

The clearance rates for HPV infection have not been extensively investigated; however, findings suggest that HPV infection clearance is quicker in men than in women [27]. Nevertheless, HR-HPV infection with the same genotype at the one-year follow-up in our population was high (71.4%), probably due to HPV16 being the most common genotype (55%) and the fact that almost 100% of female from the studies couples were infected. None of the studied risk factors were associated with the detection of HPV infection at the one-year follow-up. Detection of the same genotype at 12 months could represent either HPV persistence or a newly acquired infection. We highlight the importance of the clinical follow-up of these men, and the possible role of biopsy if visible lesions are detected.

As we reported previously [28], in our population smoking habits increased the risk of prevalent HR-HPV infection by 2.4-fold in males. These data are similar to those found in the HIM study. Smoking was previously identified as a risk factor for HPV infection in men [9,17,29]. Smoking has also been associated with HPV infection persistence, anal cancer and penile cancer in males [30] and is also a confirmed risk factor for cervical cancer risk in women [29]. At present, it is unclear how smoking may influence HPV infection in men, but several possible pathways could exist. Smoking could potentially increase the viral load by weakening cellular immune responses [31]. Since smoking cessation could be an important factor for the prevention of HPV-related diseases in both men and women, further studies focusing on the impact of smoking on HPV infection in men could be carried out.

In the present study, the prevalence of genital warts was 10.3%, which was lower than that shown in previous studies (17%) [32]. Genital warts are common in both genders and have a lower rate of occurrence in countries with a high HPV vaccination coverage [33]. The diagnosis of genital warts was the most important risk factor for HR-HPV infection in our population. Males presenting genital warts had up to 4-fold higher risk of HR-HPV infection, probably because low-risk HPV genotypes causing genital warts share transmission pathways with other HR-HPV genotypes. Co-infection with oncogenic and non-oncogenic genotypes is common [34]. A randomized, controlled trial of an adjuvanted human papillomavirus (HPV) type 6 L2E7 vaccine showed infection of external anogenital warts with multiple HPV types and failure of therapeutic vaccination, and it has also been described that patients with GW belong to a high-risk population group for HPV-related cancers [32] due to the increased likelihood of infection with oncogenic HPV genotypes.

In this study, certain sexual behaviours were associated with HR-HPV infection in men, similarly to previous reports [14,35]. For Spanish men, the average age at first sexual intercourse was 16.3 years old according to the YOURLIFE project [36]. In this study, early sexual debut (younger than 16 years old) was the third most important risk factor for HPV infection. Some authors have shown similar results [37], while other authors found different results that could be attributable not only to the age range but also to geographical characteristics [38].

Burchell *et al*. reported that 64.4% of positive subjects had ≥5 lifetime sexual partners in their population [39], which is in line with our results (54.7%). In western populations, the number of lifetime sexual partners in men and women are both relatively high, with little differences between the sexes. A higher number of sexual partners was not correlated with a higher HR-HPV prevalence in this study. This association was also absent in countries with high HPV prevalence in men such as Brazil, Colombia [13] and Mexico [38]. One explanation for this lack of association may be the geographical diversity and inaccuracy of self-reported information on sexual background. The ongoing HIM prospective multi-national study in the United States, Brazil and Mexico could clarify this issue.

Although 81.8% of HPV infected men in our population were monogamous in the previous year, this fact was not associated with higher or lower HR-HPV prevalence. In contrast, Nielson *et al*. found an association between HPV infection and number of sexual partners during the previous three months. These results might also be explained by the inclusion of low-risk genotypes in their study or by the shorter time after infection that reduced the possibility of clearing the viral infection. These two factors may have led to a higher overall prevalence.

As in other studies [28,40] we did not find evidence of a protective effect of circumcision on HPV prevalence. Similarly, in the literature it remains still unclear whether circumcision has a protective effect against HPV infection [41]. In contrast, Castellsagué *et al*. have provided epidemiologic evidence that male circumcision was associated with a reduced risk of genital infection in males [42]. Even though the protection pathway is unclear, circumcision was classically associated with the reduced persistence of HPV infection in men [43]. Removal of the foreskin could minimize the chance for new infections or could result in an increased clearance of pre-existing infections [44]. Our results could be biased because circumcision is not very common in our geographical area; thus, the statistical power to detect an association between circumcision and HPV prevalence was low. Moreover, our analysis could not assess specific associations limited to the glans penis, the area expected to be most likely protected by removal of the foreskin [45].

We did not find the expected protective effect of condom use on HPV prevalence. The effectiveness of condom use is still under discussion. Given that the virus is transmitted by skin to skin contact, condom usage has been assumed to be less effective against HPV infection compared to other sexual diseases transmitted by semen or vaginal secretions. Some authors have also reported high HPV prevalence even among those cases who report regular condom use [46]. By contrast, some studies have revealed that incorrect use of condoms increases the risk of cervical infection [35] and could be associated with a lower regression rate of cervical intraepithelial neoplasia grades 2–3 [47] in female partners.

One limitation of our study was the small sample size. Moreover, in our study, female lifestyle habits (including sexual behaviour) and histories of other sexually transmitted diseases were unknown and therefore could not be assessed. Differences between this study and previously published studies in sample collection, DNA extraction and HPV genotyping methods may also have influenced the results between studies, making it difficult to directly compare results.

Males are not only vectors but may also play a major role in the prevention of HPV-related diseases [48]. Men can acquire HPV-associated conditions and then transmit the virus to their partner and vice versa [29]. Long-term follow up may contribute to increasing knowledge of the influence of persistent HPV infection in males and potential recurrence in their sexual partners after treatment.

Although similar studies have been conducted in different countries, it is important to evaluate the different features of HPV infection in men from different populations to confirm these results.

Future trials on HPV vaccines in males should be carried out to assess the potential impact not only on the presence of penile HPV infection but also on penile subclinical lesions as a surrogate measure for the efficacy of vaccination.

## Conclusions

The presence of genital warts and smoking status seem to be associated with a higher risk of HR-HPV infection in male, and earlier sexual debut could increase this risk. Extensive knowledge of the natural history of HR-HPV infection in males could be an important goal for the design and implementation of potential prevention strategies for HPV-related disease in men, specifically for high-risk populations such as males in relationships with females after treatment for high-grade cervical lesions. Our results could contribute to improve the existing HPV prevention strategies, including targeted strategies.

## Supporting information

S1 Appendix Lifestyle questionnaire. (PDF)

S1 Table List of HPV genotypes found in multiple infection. (PDF)
