# Correction: Lifestyle factors and oncogenic papillomavirus infection in a high-risk male population

**DOI:** 10.1371/journal.pone.0197138

**Published:** 2018-05-07

**Authors:** Elena Lopez-Diez, Sonia Perez, Manuel Carballo, Amparo Iñarrea, Angel de la Orden, Maximo Castro, Moises Rodríguez, Sheila Almuster, Ruben Montero, Miguel Perez, Jorge Sanchez, Antonio Ojea

An affiliation for the first author is not indicated. Elena Lopez-Diez is also affiliated with #4, Universidade de Vigo, Vigo, Spain.
