# Supplementary material for: Lifestyle factors and oncogenic papillomavirus infection in a high-risk male population
Source: PLoS One. 2017 Sep 12;12(9):e0184492. doi: 10.1371/journal.pone.0184492 (PMC5595320; doi:10.1371/journal.pone.0184492)
Supplement: S1 Appendix — (PDF) [file pone.0184492.s001.pdf]

S1 Appendix. LIFESTYLE QUESTIONNAIRE

INFECCIÓN POR PAPILOMAVIRUS EN EL VARÓN PAREJA DE MUJER CON CIN2+

**CÓDIGO**

En este cuestionario hay ciertas preguntas que usted puede considerar de carácter íntimo, pero se pueden contestar con total sinceridad porque de ningún modo otra persona podrá saber quién contestó a este cuestionario porque se mantendrá el anonimato.

P.1. ¿En que año nació usted? 19__ __

P.2. ¿Cuál es su nacionalidad?

....

P.3. En el último año, ¿usted fumó alguna vez?

- Si

-No.

P.4. ¿A que edad empezó a fumar de forma regular?

__ __ años

No me acuerdo

P.5. Cuántos años tenía usted cuando tuvo por primera vez una relación sexual con penetración?

______ años

P.6. Ahora piense en las relaciones sexuales con penetración que tuvo usted durante toda su vida; aproximadamente ¿Cuántas parejas sexuales distintas tuvo?

Número _______

No lo sé ➔ **Aproximadamente serían……**

1
De 2 a 5
Más de 5

P.7. Ahora piense en las relaciones sexuales con penetración que tuvo usted durante el último año; aproximadamente ¿Cuántas parejas sexuales distintas tuvo?

Aproximadamente serían……

1
De 2 a 5
Más de 5

P.8.- ¿Ha utilizado condón en todas sus relaciones sexuales durante el último año?

-Si

-No

PAPILLOMAVIRUS INFECTION IN MALE SEXUAL PARTNERS OF WOMEN DIAGNOSED OF HIGH GRADE CERVICAL LESIONS

Code

Some questions in this questionnaire can be considered as intimate. Your anonymity will be kept so you can answer these questions with total sincerity.

P.1. What year were you born? 19___

P.2. What is your nationality?

....

P.3. In the last year, have you ever smoked?

- Yes
- No

P.4. How old were you when you started smoking on regular basis?

__ __ years old

I don`t remember

P.5. How old were you when you had your first sexual intercourse with penetration?

______ years old

P.6. **Now think about sex with penetration that you had throughout your life; How many different sexual partners did you have?**

Number _______

I don't know exactly ➔ Approximately……

1
Between 2 and 5
More than 5

P.7. Now think about sex with penetration that you had during the last year; How many different sexual partners did you have?

Approximately…

1
Between 2 and 5
More than 5

P.8. **Have you used condoms in every sexual intercourse during the last year?**

-Yes

- No
