# Supplementary material for: Lifestyle factors and oncogenic papillomavirus infection in a high-risk male population
Source: PLoS One. 2017 Sep 12;12(9):e0184492. doi: 10.1371/journal.pone.0184492 (PMC5595320; doi:10.1371/journal.pone.0184492)
Supplement: S1 Table — (PDF) [file pone.0184492.s002.pdf]

S2 Table. List of HPV genotypes found in multiple infection

	**Number of Samples**			
**HPV Genotype**	**2 genotypes**	**3 genotypes**	**4 genotypes**	**5 genotypes**
**6**	2	2	3	1
**16**	14	5	5	4
**18**	3		1	
**26**	1			
**31**	4	1	1	2
**33**	1			1
**35**	2			
**39**	1	1	1	
**45**		1	2	1
**51**	5	2	1	4
**52**	1	2	3	2
**53**	5	2	3	2
**56**	1	3	3	2
**58**	1	2		
**59**		1	3	3
**66**	3		2	3
**67**			1	
**68**				2
**69**		1		
**70**	1	1		1
**73**	2		2	1
